# Characterisation of influenza A viruses with mutations in segment 5 packaging signals

**DOI:** 10.1016/j.vaccine.2009.05.053

**Published:** 2009-10-23

**Authors:** Edward C. Hutchinson, Helen M. Wise, Katerine Kudryavtseva, Martin D. Curran, Paul Digard

**Affiliations:** a Division of Virology, Department of Pathology, University of Cambridge, Tennis Court Road, Cambridge CB2 1QP, United Kingdom; b Health Protection Agency, Clinical Microbiology and Public Health Laboratory, Addenbrooke's Hospital, Hills Road, Cambridge CB2 2QW, United Kingdom

**Keywords:** Influenza, Packaging signal, Nucleoprotein

## Abstract

Influenza A virus vRNA segments contain specific packaging signals at their termini that overlap the coding regions. To further characterise segment 5 packaging signals, we introduced synonymous mutations into the terminal coding regions of the vRNA and characterised the replicative fitness of the resulting viruses. Most mutations tested were well-tolerated, but a virus with alterations to NP codons 464-466, near the 5′-end of the vRNA, produced small plaques and replicated to around one-tenth of the level of wild type virus. The mutant virus supported normal levels of NP and segment 5 vRNA synthesis but packaged reduced levels of both segment 5 and segment 3 into virus particles. This suggests an interaction between segments 3 and 5 during influenza A virus assembly.

## 1 Introduction

The negative-sense RNA of the influenza A virus genome is divided into eight segments, which are packaged into new virions as they assemble at the plasma membrane of infected cells. As influenza virions do not typically package more than eight segments in total [1–5], packaging a random selection of segments would result in an extremely small proportion of new virions having the full complement of genes required to initiate further infections [3]. As a result, the virus has evolved a mechanism to help ensure each of its eight segments is selectively packaged [6,7]. This selective packaging mechanism utilises *cis*-acting RNA packaging signals in each of the eight segments, the location of which has been inferred from the structure of defective interfering (DI) RNAs [6,8], the packaging of recombinant virion RNA (vRNA) molecules [7,9–13] and the conservation of primary nucleotide sequences [14–16]. Additionally, reverse genetics has been used to introduce point mutations into segments 1–4, 7 and 8, identifying nucleotides that contribute to *cis*-acting RNA signals, including those required for efficient genome packaging [9,10,13–18].

Despite the identification of packaging signals in each of the eight segments of the genome, their mechanism of function remains obscure. A favoured hypothesis is that each segment interacts with a particular set of neighbouring segments, most likely through RNA–RNA interactions, to assemble a specific ‘genome complex’ containing all eight segments [7,19]. This is consistent with ultrastructural studies showing a complex of eight parallel and closely aligned segments at the point of viral assembly and within virions [1,4,5], as well as with studies in which mutational disruption of the packaging of particular segments affected the incorporation of various other segments in *trans* [10,15,16,18,20]. However, this hypothesis predicts a specific array of vRNAs within the virion and the order of segments in this putative genome complex is currently unknown. This uncertainty, along with the complex tertiary structure adopted by each vRNA when it is folded and encapsidated to form a ribonucleoprotein complex (RNP), has made the identification or prediction of specific interactions between the segments difficult [14,21]. One way to address this problem is to use viral reverse genetics in order to identify *trans*-interacting sequences on the various segments.

We have previously used a bioinformatics approach to identify regions of the eight vRNA coding regions under selection pressure for primary RNA sequence as well as their coding capacity [14]. Regions of low synonymous codon variation correspond to the location of *cis*-acting RNA sequences and have been successfully used to guide the mutation of packaging signals in segments 1, 6 and 7 [14,16,18]. In this paper, we extend this approach to segment 5 of influenza A virus. We found that segment 5 packaging signals were less sensitive to mutational disruption than those of segment 7. However, one cluster of mutations (in codons F464-L466) significantly affected virus fitness by disrupting packaging of segment 5 and, interestingly, also segment 3.

## 2 Materials and methods

### 2.1 Cells, virus, plasmids and antisera

Human embryonic kidney 293T cells and Madin-Darby canine kidney (MDCK) cells were cultured as described previously [22]. Influenza A/PR/8/34 (PR8) virus was generated using an eight-plasmid reverse genetics system kindly donated by Professor R. Fouchier [23]. Site-directed mutagenesis of the reverse genetics plasmids was carried out using mismatched PCR primers and native PFU polymerase (Stratagene). Plasmids were sequenced using a combination of terminal primers and (where necessary) internal primers by the University of Cambridge Department of Biochemistry sequencing facility. Primers and PCR conditions are available on request. Plasmids pCDNA-PB2, pCDNA-PB1, and pCDNA-PA have been described previously [24]. Plasmid pHumanPolI-ffLuc was a kind gift of Dr L. Tiley [25]. Rabbit anti-NP and anti-M1 sera have been described previously [26,27]. Secondary antibodies were purchased from Molecular Probes or LiCor Biosciences (fluorescent conjugates) or DAKO (horseradish-peroxidase conjugates).

### 2.2 Reverse genetics and virus titrations

Recombinant viruses were produced by transfection of 293T cells with the reverse genetics plasmids, and working stocks produced by subsequent infection of MDCK cells for 48 h, as described previously [18]. Additional stocks were produced by infecting eight-day-old embryonated chicken eggs for 48 h [18]. Segment 5 from all stocks of virus was sequenced to confirm the presence of the desired mutations. RNA was extracted from infected cells using the SV Total RNA isolation system (Promega) or from virus stock using Tri Reagent LS (Sigma), reverse transcribed using avian myeloblastosis virus reverse transcriptase (Promega) and a terminal vRNA-binding primer, and amplified by PCR using terminal primers and Illustra *Taq* DNA polymerase (GE Healthcare). Primers and reaction conditions are available on request. Plaque assays were carried out on confluent MDCK cells; plaque assays and plaque size analysis were performed as described in [18].

### 2.3 Protein and RNA analyses

Infected cell lysates were analysed by SDS-PAGE and western blotting according to standard procedures. Blots were imaged by chemiluminescence using horseradish-peroxidase conjugated secondary antibodies and X-ray film, or by fluorescence using IRDye 800 conjugated secondary antibodies on a Li-Cor Biosciences Odyssey near-infrared imaging platform. To examine the protein content of virus particles, virus stocks were cleared of debris by low-speed centrifugation and then pelleted through a cushion of 33% sucrose in PBS at 91,000 × *g* for 45 min at 4 °C, as described elsewhere [18]; pellets were resuspended in 20 μl SDS-PAGE sample buffer. Reverse transcription to detect segment 5 or 7 vRNA in 500 ng of total RNA extracted from infected cells 8 h p.i. was carried out using avian myeloblastosis virus reverse transcriptase (Promega) and a terminal vRNA-binding primer (5′-AGC GAA AGC AGG AGT TTA AAA TG). Aliquots of these reactions were used in 25 cycles of PCR with Illustra *Taq* DNA polymerase (GE Healthcare) and terminal vRNA- and cRNA-binding primers (as above, and 5′- AGT AGA AAC AAG GAG TTT TTT GAA CAG, respectively); reaction conditions are available on request. The total vRNA content of virus particles was analysed by 6% urea-PAGE and silver staining as previously described [18]. Densitometry was carried out using the program ImageJ [28]. Quantitative RT-PCR was performed essentially as previously described [18] using the SuperScript III Platinum one-step qRT-PCR system (Invitrogen) and a Rotor-Gene 3000 real-time thermal cycler (Corbett Research Limited), using protocols based on UK National Standard method VSOP 25 (www.hpa-standardmethods.org.uk). Reaction conditions, primers, and TaqMan probe sequences are available upon request.

### 2.4 RNP reconstitution assay

1 × 10^6^ 293 T cells per 35 mm well were transfected in suspension using Lipofectin (Invitrogen). To reconstitute RNPs, 250 ng each of pCDNA-PB2, pCDNA-PB1, and pCDNA-PA were transfected along with 250 ng of an NP-expressing reverse genetics plasmid and 100 ng of pHumanPolI-ffLuc. Following incubation at 37 °C for 48 h, cells were lysed and luciferase levels determined with an AutoLumat LB953 luminometer (EG&G Berthold), using 0.6 mM beetle luciferin (Promega).

## 3 Results

### 3.1 Mutation of conserved codons in the terminal regions of segment 5

**Fig. 1:**
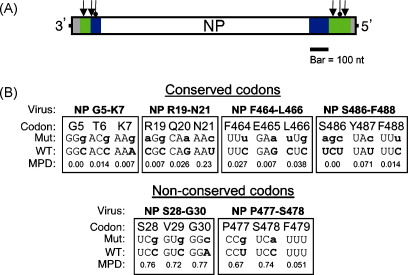
Mutation of putative *cis*-acting signals in segment 5. (A) Scale diagram of segment 5 vRNA, showing non-coding regions in grey, and the coding region in white. Areas of the coding region defined as the minimal regions required for efficient packaging of a reporter construct are shown in green [12] while additional coding regions present in the shortest reported DI RNA are shown in blue [29]. Pointed arrows indicate mutations introduced to conserved codons and round-headed arrows indicate mutations in non-conserved codons. (B) Details of mutations. For each altered codon the mutant (mut) and wild type (WT) nucleotide sequences are shown in coding sense, with mutations indicated by lowercase bold letters. Also shown is the mean pairwise distance (MPD) score of each codon, scaled from 0 (absolutely conserved) to 1 (no conservation beyond that expected from amino acid constraint) [14](For interpretation of the references to colour in this figure legend, the reader is referred to the web version of the article.).

Previously, we showed that the introduction of synonymous mutations into normally highly conserved codons in the terminal regions of segments 1, 6 and 7 caused defects in vRNA incorporation into virus particles, consistent with the presence of *cis*-acting packaging signals [14,18]. Here, we applied the same techniques to study *cis*-acting RNA signals in segment 5 of the virus. The structure of a DI RNA derived from segment 5 [29], our previous bioinformatics analysis [14], as well as the minimal segment 5-derived sequences required to package a reporter gene [12] indicated that, similarly to the other segments of the influenza A genome, the terminal regions of segment 5 vRNA contained packaging signals (Fig. 1A). However, to the best of our knowledge, the structure/function of these packaging signals had not been previously investigated experimentally at the nucleotide level.

**Fig. 2:**
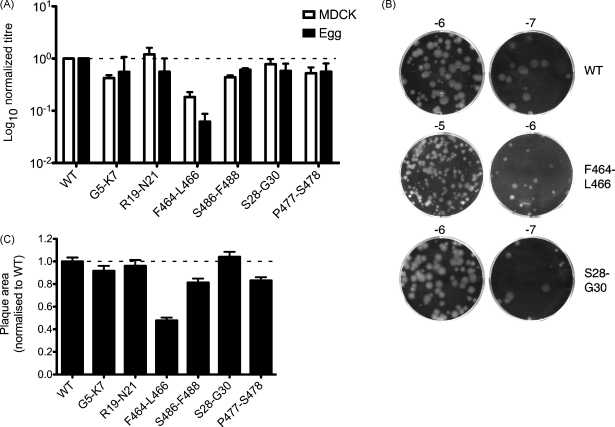
Growth characteristics of segment 5 mutant viruses. (A) Growth characteristics of WT and mutant viruses. MDCK cells or embryonated eggs were infected at low multiplicity and virus titres at 48 h p.i. determined by plaque assay in MDCK cells. Values are plotted relative to the titre achieved by the WT virus in each experiment. The mean + half-range of two isolates are shown for egg-grown viruses while the mean + SEM of between 2 and 9 isolations are plotted for MDCK-grown viruses. (B) Representative images of plaques formed by MDCK-grown viruses. Log_10_ of the concentration of virus stock used is shown above each well. (C) Plaque areas of viruses from MDCK- and egg-grown stocks were quantified and normalised with respect to the mean area of WT virus prepared under the same conditions. Between 139 and 287 plaque areas were measured for each virus; mean + SEM values are shown.

Accordingly, guided by our previous bioinformatics analysis [14], clusters of three adjacent, highly conserved codons were identified in the terminal coding regions of segment 5 and the maximum number of synonymous mutations (3–5 per mutant) introduced into a reverse genetics cDNA clone of the PR8 segment (Fig. 1B). Additionally, we noted that codons are found throughout the influenza genome which, though variable, have only been recorded as utilising a subset of possible nucleotide sequences (supplementary data of [14]). We identified two clusters of codons in which the third ‘wobble’ base of non-conserved codons was strongly biased towards usage of either purines (G30) or pyrimidines (S28, V29, P477 and S478). To test whether this reflected a functional role, we introduced synonymous mutations that violated this bias (Fig. 1B). WT and mutant viruses were rescued by transfection of plasmids encoding all eight segments of the viral genome into 293 T cells. Independent rescues were carried out between two and nine times, depending on the virus. The growth parameters of the mutant viruses in MDCK cells were then characterised. Despite carrying mutations not normally observed in segment 5, the majority of viruses grew almost as well as the WT virus, reaching average titres of more than 1 × 10^8^ PFU/ml (Fig. 2A). However, the virus F464-L466 produced on average, titres that were more than 5-fold lower than the WT virus (Fig. 2A). To measure growth properties in a more physiological system, aliquots of MDCK cell-grown viruses were used to inoculate embryonated chicken eggs. In this system, most viruses also yielded similar plaque titres to WT, with an overall average titre of 1.8 × 10^9^ PFU/ml. However, the F464-L466 virus again showed a replication defect, producing titres that were more than 10-fold lower than those of WT virus (Fig. 2A). It was also noticeable that the plaques formed by the F464-L466 virus in MDCK cells were generally smaller than those of the WT virus or the other mutant viruses (Fig. 2B and data not shown). In confirmation of this, when plaque areas of the viruses (whether MDCK-grown or egg-grown) were measured, most viruses produced plaques with an average area similar to WT, but plaques from the F464-L466 virus were less than half the area of the wild type virus (Fig. 2C). Overall therefore, most of the synonymous mutations chosen to probe the function of the segment 5 packaging signals had only minor (less than 2-fold) effects on virus replication. However, a virus with mutations in NP codons F464-L466 showed a more substantial reduction in growth properties.

### 3.2 Effects of synonymous mutations on viral macromolecular synthesis

**Fig. 3:**
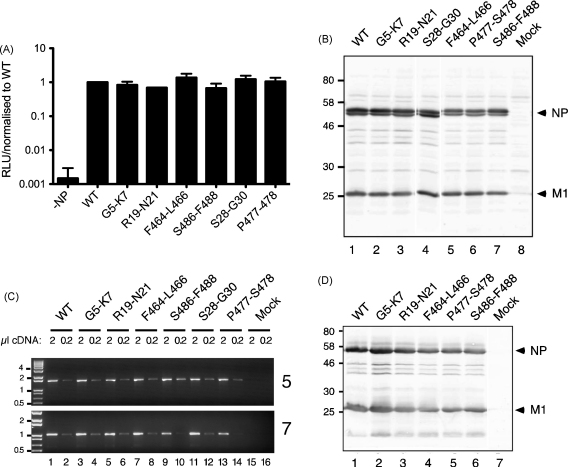
Effect of mutations on viral macromolecular synthesis. (A) Ability of NP encoded by the mutant segments to promote viral gene expression. RNPs containing a synthetic vRNA encoding luciferase were reconstituted in 293T cells by transfection, with NP provided by the reverse genetics plasmids used to generate the mutant viruses (−NP; NP omitted as a negative control). Luciferase activity in cell lysates was determined 48 h post-transfection. Data are the mean + half-range of two experiments except for R19-L21 (a single experiment) and P477-S478 and S486-F488 (mean + half-range of three experiments). (B) NP and M1 expression in infected cells. MDCK cells were infected at an MOI of 1 and at 18 h p.i., cell lysates analysed by SDS-PAGE and western blotting. The migration of NP, M1 and molecular mass standards (kDa) are indicated. (C) vRNA accumulation in infected cells. MDCK cells were infected at an MOI of 3 and RNA isolated at 8 h p.i. 500 ng of total RNA was reverse transcribed and fixed volumes of the resulting cDNA (as indicated) used as PCR templates for amplification of full-length segment 5 or 7. PCR products were analysed by agarose gel electrophoresis and ethidium staining. Selected size markers (kb) are indicated. (D) NP and M1 content of egg-grown virus particles. Virions from equal volumes of allantoic fluid (corresponding to 3.9 × 10^8^ PFU of WT virus), were pelleted through a sucrose pad before analysis by SDS-PAGE and western blotting.

Segment 5 encodes NP, a multifunctional protein with important roles in viral RNA synthesis and virion assembly [21,30]. Synonymous mutations are unlikely to affect protein function, but to test whether the NP proteins encoded by the mutated segments were still active in viral gene expression, their function was tested in RNP reconstitution assays. Cells were transfected with plasmids that expressed a synthetic vRNA containing (in antisense) a luciferase reporter gene, the WT viral polymerase proteins and plasmids encoding either WT or mutant NP genes. The resulting luciferase expression was then quantified as a measure of the ability of the various NP mutants to support viral gene expression. All segment 5 clones, whether WT or mutant, produced NP that promoted viral transcription to similar levels, approximately 1000-fold higher than a negative control lacking NP (Fig. 3A). Similarly, when viral gene expression was examined in infected MDCK cells, all viruses produced comparable amounts of both NP and the matrix protein M1 (Fig. 3B). Next, we examined vRNA accumulation in infected cells, as several previous studies have found that mutations in the terminal regions of the segments outside of the core promoter sequences can affect vRNA expression [14,18,31,32]. To this end, RNA isolated from infected cells was analysed by RT-PCR for vRNA from segments 5 and 7. However, similar amounts of cDNA product were produced for both segments from all viruses (Fig. 3C). Seeding of the PCR reactions with 10-fold dilutions of the RT products confirmed that the assay was working in the semi-quantitative range (e.g. compare lanes 1 and 2), while no products were detected in reactions in which either the RT or the RT primer was left out (data not shown), confirming the strand specificity of the RT-PCR as well as the lack of any signal from potential carry-over of the reverse genetics plasmids. Similar results were obtained from a replicate experiment using independently isolated virus stocks (data not shown). Thus, the synonymous mutations introduced into segment 5 do not appear to affect viral macromolecular synthesis, even in the case of the poorly replicating F464-L466 virus.

### 3.3 Characterisation of a segment 5 packaging defect

Next, we investigated whether the replication defect of the F464-L466 virus resulted from impaired genome packaging. Previously, we found that mutation of the segment 7 packaging signals lead to lower overall incorporation of NP/RNPs into virus particles when the mutant viruses were grown in eggs [18]. However, when the NP and M1 content of the segment 5 mutant viruses was examined by western blotting of virions pelleted through a sucrose pad, comparable amounts of protein were found in all viruses, including F464-L466 (Fig. 3D).

**Fig. 4:**
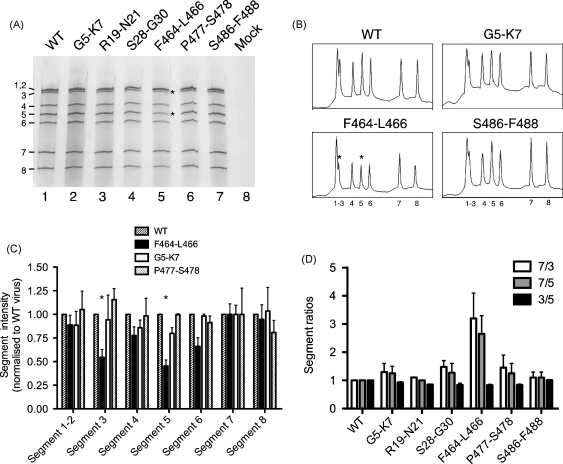
Effect of mutations on vRNA packaging. (A) vRNA was extracted from equal titres of virus (corresponding to 1.4 × 10^8^ PFU/lane) and analysed by urea-PAGE and silver staining. Segment numbers are indicated. Asterisks highlight under-represented vRNA species in F464-L466 virus. (B) Densitometry traces of selected lanes from (A), with reduced peaks indicated by asterisks. The segments corresponding to each peak are given beneath the traces. (C) Densitometric quantification of virion RNA content. Three separate preparations of vRNA from two independently rescued virus stocks were analysed as in (A) and (B). For every mutant the area of each peak was expressed relative to the total peak area for that virus (to account for small errors in input PFU) and was then normalised to segment 7. Mean + SEM values are plotted with asterisks indicating the reduced segments 3 and 5. (D) Relative incorporation of specific segments into the virus population. The copy number of segments 3, 5 and 7 from equal PFU of the viruses was determined by qRT-PCR and normalised with respect to WT virus for each segment. The ratios of the indicated pairs of segments are plotted (mean + half-range from measurements of two independent stocks of virus).

Although the presence of approximately equal levels of NP in the virions suggested that the mutations did not alter overall levels of genome packaging, it remained possible that the packaging of specific segments was affected. To investigate this, vRNA was extracted from equal quantities of virus (according to plaque titre) and analysed by urea-PAGE and silver staining. RNA species of sizes expected for the eight segments were clearly observed in samples from both mutant and WT viruses, with no staining detectable in a sample of mock-infected allantoic fluid prepared in the same manner (Fig. 4A; note that under these conditions segments 1 and 2 co-migrate). Overall, the mutant viruses packaged similar amounts of vRNA to WT, in agreement with the levels of NP incorporation. However, close inspection of the gel suggested there were reduced amounts of segments 3 and 5 in the F464-L466 virus compared to WT (Fig. 4A, lane 5, highlighted by asterisks). Densitometric analysis of the gel confirmed this observation, as samples from WT and all mutant viruses other than F464-L466 produced traces in which the peaks corresponding to segment 5 were distinctly higher than the flanking segments 4 and 6, while segment 3 formed a subsidiary peak only slightly lower than the co-migrating segments 1 and 2 (Fig. 4B and data not shown). In contrast, the peak heights of segments 3 and 5 in the trace from virus F464-L466 were noticeably lower. When the areas under each peak were calculated from three replicate analyses, segments 3 and 5 were consistently around 2-fold lower in F464-L466 compared to WT (Fig. 4C), while segments in the other viruses were similar to WT. This suggested that F464-L466 did indeed possess a specific packaging defect.

To further examine vRNA packaging in the mutant viruses, the relative amounts of segments 3, 5 and 7 incorporated into the viruses was measured by qRT-PCR. In most viruses the ratios of these segments to each other were similar to those of WT, fluctuating around 1:1 values (Fig. 4D). However, the ratios of segment 7 (whose packaging did not appear by silver stain to be reduced) to segments 3 and 5 in virus F464-L466 were increased by around 3-fold (Fig. 4 D), consistent with the under-representation of the latter two segments seen by direct PAGE analysis of the vRNAs (Fig. 4C). The ratio of segments 3 to 5 was not affected in this virus (Fig. 4D), suggesting that incorporation of both segments into F464-L466 virions was reduced to an approximately equal extent. Thus, the introduction of synonymous mutations into NP codons F464-L466 results in a specific packaging defect affecting the incorporation of segments 3 and 5.

## 4 Discussion

By using a prior bioinformatics analysis of codon variation in influenza A virus [14] we set out to better define the packaging signals in segment 5 of influenza A virus. We targeted codons with low variability or whose variability appeared to be constrained to either purine or pyrimidine usage for mutational analysis. The maximum number of novel synonymous mutations were introduced in clusters of 2 or 3 adjacent codons, without substantially increasing the usage of codons that were rare in the genomes of influenza A [14], dogs or chickens (http://www.kazusa.or.jp/codon/), or of codon pairs that are uncommon in the human genome [33]. All mutations lay in regions retained by a segment 5-derived DI RNA [29] and all except S28-G30 lay within the minimal flanking regions required for efficient packaging of a reporter gene [12]. None of the mutations characterised here appeared to significantly affect the function of the segment 5 gene product, NP, or viral macromolecular synthesis in general. When NP transcriptional function was tested, the mutants showed activities within 1.5-fold of the WT protein (Fig. 3A) and when accumulation of NP was measured, again, no more than a 1.5-fold deviation from the WT value was seen (data not shown). Indeed, most of the mutations had little overall effect on virus growth, with 5 of the 6 mutant viruses replicating to very similar levels as the WT virus and only one virus (F464-L466) showing a relatively modest fitness decrease of around 10-fold. This contrasts with the outcome when we applied a very similar strategy to segment 7, where all viruses with lesions in conserved regions of the packaging signals showed growth defects of between 10 and 1000-fold relative to WT virus [18]. One possibility is that the mutations chosen in this study were simply ‘unlucky’ and did not target crucial residues, or a sufficient number of residues, to prevent function of the packaging signal. This is consistent with the recent work showing that the packaging signals of segments 7 and 8 can accommodate a diverse range of mutations without major effects on packaging [13,17], and with a study in which a group of mutations in the terminal regions of segment 3 that could not be rescued together caused minimal defects when mutated as two separate groups [16]. An alternative possibility is that the segment 5 packaging signals are simply more tolerant of mutation than those in segment 7, perhaps because the latter segment plays a more important role in directing the incorporation of other segments [18]. A better understanding of how packaging signals function is required to distinguish between these hypotheses.

Nevertheless, one of the mutants in this study, F464-L466, did display a phenotype consistent with a segment-specific packaging defect. While we cannot categorically rule out defects in the intracellular stages of its life cycle, the most obvious defects were seen in the vRNA content of released particles. As well as showing a 2–3-fold deficiency in packaging of the mutated segment 5, F464-L466 also showed a comparable reduction in packaging of the WT segment 3 (Fig. 4C and D). This further suggests that during virion assembly, segment 5 interacts, directly or indirectly, with segment 3. It is interesting to note a similar reduction in the packaging of segment 5 has been reported following the introduction of between 6 and 14 synonymous point mutations in the 5′-end of segment 3 vRNA [16]. Further work is required to investigate whether this indicates a reciprocal interaction between these sites during packaging, but inter-segment interactions of this sort are predicted by the current model of packaging involving the assembly of a ‘genome complex’ stabilised by RNA–RNA interactions between the eight vRNAs. Thus although the packaging mechanism of influenza A virus remains unclear, further characterisation of the RNA signals at the nucleotide level may begin to provide clues to its mode of action.
